# Chronic graft versus host disease in upper limb: Calling upper limb rehabilitation specialists for improving outcomes in patients

**DOI:** 10.46989/001c.158500

**Published:** 2026-03-11

**Authors:** Jaleel A. Mohammed, Caroline Couthard, Ahmed AlGhamdi, Collins Ogbeivor, Shahrukh K. Hashmi

**Affiliations:** 1 Physical Therapy Department, King Faisal Specialist Hospital & Research Centre, Riyadh, KSA; 2 European Society for Shoulder and Elbow Rehabilitation (EUSSER), Geneva, Europe; 3 University of Hertfordshire, Hatfield, Hertfordshire, AL10 9AB; 4 Division of Hematology, Department of Medicine, Mayo Clinic, Rochester, MN, USA

**Keywords:** graft versus host disease, musculoskeletal, physiotherapy, occupational therapy, rehabilitation

## Abstract

**Background:**

Chronic graft versus host disease (cGVHD) is a complex post-transplant complication resulting in varying degrees of musculoskeletal (MSK) manifestations, including sclerosis-type skin conditions, fasciitis, and osteopenia, avascular necrosis affecting the shoulder, elbow and hands apart from other joints of the body. A large proportion of these can often miss out on the expertise of upper limb therapists.

**Study Objectives:**

This paper aims to outline upper limb cGVHD manifestations to raise awareness among upper limb physiotherapists and rehabilitation specialists, so that patients can receive more focused, optimal care.

**Methods:**

An electronic search was conducted across a wide database covering the period from 1990 to 2025. Further manual search used Google Scholar to help identify relevant articles. Boolean operators ‘AND’ and ‘OR’ combined keywords such as chronic graft versus host disease, acute graft versus host disease, allogenic hematopoietic stem cell transplantation, hematopoietic cell transplantation, stem cell transplant, bone marrow transplant, physiotherapy, upper limb specialist, musculoskeletal, fasciitis, and scleroderma.

**Results:**

A total of 4,393 titles were screened against the pre-set inclusion and exclusion criteria, and only 3 articles were found to be suitable for this narrative review. All 3 articles were case studies of upper limb cGVHD and rehabilitation reporting positive outcomes on patients’ upper and lower limb range of motion, function, and quality of life (QoL).

**Conclusion:**

cGVHD can cause various MSK issues, especially in the upper limbs. It is important to collaborate with upper limb physiotherapy and occupational therapy experts to improve awareness and patient outcomes.

## Background

Chronic graft versus host disease (cGVHD) following allogeneic hematopoietic cell transplantation (allo-HCT) is a complex and multifaceted syndrome that can present with a wide range of manifestations, impacting up to 70% of patients.^1^ The clinical manifestations of cGVHD can involve multiple organs, including soft tissue, skin, mouth, eyes, gastrointestinal (GI) tract, liver, lungs, joints, muscles, cardiovascular system, and sexual organs.^2–4^ The burden of this disease can significantly affect patients’ quality of life (QoL), activities of daily living (ADL), and function, and can psychologically influence their lifestyle, social interactions, and ability to resume normal daily activities.^5^ Among the joints and fascia affected by cGVHD, the wrist and fingers are reported to be the most frequently involved, resulting in grip weakness, wrist and finger joint contractures, hand muscle atrophy, and peripheral neurological symptoms.^6,7^ To help mitigate these long-term effects, the American Society for Transplantation and Cellular Therapy (ASTCT) practice guideline committee has proposed recommendations that emphasize the importance of long-term outpatient follow-up for HCT recipients, including physiotherapy, early detection services, and expert management of late complications.^8^

Although physiotherapy and occupational therapy have been recommended for managing musculoskeletal (MSK) manifestations in cGVHD by numerous experts and consensus groups, the treatment interventions used vary significantly across HCT units and countries. This variability may be attributed to challenges clinicians face, including a limited understanding of the disease process and its underlying pathophysiology. Furthermore, in our observation in a typical outpatient MSK setting, therapists who are not part of an HCT multidisciplinary team have little exposure to this patient population and may therefore be less aware of the complexities associated with cGVHD, including the subtleties of the disease progression and MSK manifestations, which can lead to suboptimal care.

## Aims

The primary aim of this paper is to review and synthesize the current evidence base on MSK complications of cGVHD affecting the upper limb (UL). This will be used to address the key objectives of the study:

1. Set the background for the development of GVHD as a side effect of allo-HCT
2. Outline and describe the MSK complications that affect the UL caused by cGVHD.
3. Make clinical practice recommendations to UL rehabilitation specialists, including physiotherapists and occupational therapists, to optimize patient care.
4. Consider suitable strategies to raise awareness of this condition amongst UL rehabilitation specialists to mobilize knowledge.

Although there is paucity of published literature specifically addressing MSK cGVHD manifestations in the UL from the rehabilitation perspective, we have endeavored to compile relevant information to serve as a preliminary guide for UL clinicians.

## Methods

For this narrative review, an electronic search was conducted using MEDLINE/PubMed, Scopus, Web of Science, and Embase databases covering the period from 1990 to 2025 (Appendix I). Further manual search was conducted using Google Scholar. For a comprehensive search, the Boolean operators ‘AND’ and ‘OR’ were used using keywords chronic graft versus host disease, acute graft versus host disease, allogenic hematopoietic stem cell transplantation, hematopoietic cell transplantation, stem cell transplant, bone marrow transplant, physiotherapy, upper limb specialist, musculoskeletal, fasciitis, and scleroderma. A total of 4,393 titles were screened against the pre-set inclusion and exclusion criteria (Table 1). Text, word, and thesaurus searches were used to minimize the risk of missing relevant articles. The reference lists of identified articles were manually searched for additional relevant references.

**Table 1.** Inclusion and Exclusion Criteria

Inclusion	Exclusion
Studies included hematopoietic stem cell transplant patients, graft versus host disease patients, patients with post-transplant musculoskeletal manifestations, scleroderma, fasciitis. Studies in English language Publications between 1990 – 2025	Studies not in English Non-⁠musculoskeletal GVHD manifestations

## Results

A total of 4,393 titles were screened against the pre-set inclusion and exclusion criteria, and only 3 articles were found to be suitable for this narrative review. Of these, Lemes et al. (2025)^9^ full text article was not available and it appears to be a conference abstract. The other 2 articles were case studies of patients with UL cGVHD and show that early intensive rehabilitation coupled with regular home exercises are key for managing its symptoms. Table 2 outlines the findings from the case studies.

**Table 2.** Shortlisted case studies

Article	No. of Patients	Upper limb GVHD	Intervention	Outcomes
Jung et al (2019)^10^	6 Children	Scleroderma like changes, tightening, fibrosis, and loss of elasticity in the skin and underlying fascia, with joint contractures due to skin/fascial tightening in shoulders, elbows, wrists, and fingers.	Physical therapy, occupational therapy and education to parents on home exercises	Only three of the six children showed improved range of motion (ROM). Better outcomes were linked to starting treatment within 2 months of cGVHD onset and completing home-based exercises twice daily for 30 minutes. Delayed treatment (after 6 months) and poor exercise compliance resulted in less improvement.
Tendas et al (2011)^11^	1 Adult	Extensive skin and musculoskeletal cGVHD with severe reduction of joints ROM of bilateral shoulders, elbows and hips, and also bilateral legs muscles hypotrophy due to spinal cord disease-related compression.	Motor rehabilitation, physical and occupational therapy with particular attention to stretching exercises	The patient showed 50% improvement in ROM, along with gains in motor skills, psychology, and QoL after 4 weeks. However, treatment was halted at 5 weeks due to pneumonia, causing rapid deterioration.

## Introduction of HCT to UL MSK Practitioners

HCT is increasingly being used for treating various malignant and non-malignant diseases, and solid tumors.^12,13^ Over the past few decades, the use of HCT has seen a significant rise globally, with an increase from 46,563 procedures in 2006 to 82,718 in 2016, representing a 77.6% growth.^14^ The first two years after HCT carry a high risk of mortality, but survival improves significantly thereafter. Patients in remission at two years have an estimated 10-year overall survival rate of up to 90%, reflecting a threefold increase in survivorship over the past 25 years.^15–17^ However, despite improved survival rate, many patients experience a diminished quality of life (QoL), loss of income and work, and greater reliance on caregivers due to disease and treatment-induced complications.^18–20^

## Types of HCT

There are two primary types of HCT transplants, autologous and allogeneic. Autologous transplants (auto-HCT) use the patient’s own stem cells, whereas allo-HCT involves a donor.^21^ The selection of donor cells utilized for transplantation is contingent upon a multitude of factors, including the availability of donors, the specific type of malignancy, and the objectives of the treatment. The donor cells designated for transplantation are generally sourced from three discrete origins: the bone marrow (BM), frequently referred to as a BM harvest; the peripheral circulation, identified as a peripheral blood stem cell (PBSC) collection; and, in exceptional cases, from blood procured from the umbilical cord (UCB) subsequent to the delivery of a healthy neonate, which is typically preserved in UCB repositories.^22^

## Impact on QoL

HCT patients are faced with various challenges during their course of treatment and recovery, which can include social withdrawal, reduction in ADL due to related disability, increased reliance on carers, loss of income, and family breakdown.^23,24^ Furthermore, these repercussions extend beyond physical challenges; they also encompass significant psychological and socioeconomic consequences due to additional financial burdens and unemployment. Adverse psychosocial outcomes, elevated depression and anxiety levels, and, in some instances, risk of suicidal thoughts and deaths have been reported in the literature.^25–27^

## GVHD

One of the major complications post-allo-HCT is GVHD, in which donor graft T-lymphocytes recognize the recipient’s tissues as foreign, leading to an immune-mediated attack on the host.^28^ GVHD is classified as acute and chronic and was previously distinguished by the respective timelines of occurrence: aGVHD was thought to occur when symptoms appeared within the first 100 days of transplantation, whereas cGVHD was defined as manifestations that occurred after that time.^29^ However, advances in understanding the disease process led to the introduction of the overlap syndrome concept, as proposed by the 2005 National Institutes of Health (NIH) consensus.^30^

cGVHD is recognized as a distinct syndrome that may overlap with aGVHD, with features of both potentially present simultaneously. It is not defined solely by onset after day 100; it can arise earlier, evolve from aGVHD, or occur de novo, with diverse clinical manifestations.^31,32^ The incidence of aGVHD in HCT patients has been reported to be as high as 50%, while cGVHD can affect up to 80% of patients.^33^

## MSK Manifestations of cGVHD

cGVHD affects multiple organs and can have a significant, far-reaching impact on an individual’s health. The MSK manifestations of cGVHD include osteoporosis, mechanical and inflammatory arthralgia, synovitis, tenosynovitis, fasciitis, myopathy, sclerodermatous contractures, peripheral neuropathy, and physical deconditioning.^34,35^ The fasciitis, scleroderma, and joint involvement often pertain to the upper limbs, significantly affecting the hands and fingers, resulting in weak grip strength, reduced range of motion (ROM) in shoulders, elbows, wrists, and fingers, and reduced functional capacity resulting in disability.^36–38^

The complex nature of this disease is further heightened by the fact that certain cGVHD manifestations can resemble other musculoskeletal conditions, for example:

- Features of connective tissue disorders, inflammatory changes, and extra-glandular symptoms that are also characteristic of primary and secondary Sjögren’s syndrome.^39^
- A similarity to rheumatoid arthritis (RA) is marked by clinical signs and early radiological findings indicative of RA, elevated rheumatoid factor (RF), and rapid progression of symmetrical joint space narrowing in the knees and wrists within a year, as well as atlantoaxial subluxation (C1-C2).^40^
- Pain, proximal muscle weakness, and muscular hypotrophy as seen in polymyositis with positive findings from electromyograms (EMG) and muscle biopsies, positive HLA DR52, and elevated levels of creatine phosphokinase (CPK), aldolase, and serum glutamic pyruvic transaminase (SGPT).^41^

## Skin cGVHD^42,43^

Cutaneous cGVHD affects up to 75% of patients with cGVHD and is a major cause of morbidity, pain, restricted ROM, resulting in contractures, disability, and loss of function. Its clinical presentation is heterogeneous and often mimics various autoimmune and autoinflammatory dermatoses. The spectrum of disease covers the non-sclerotic form, historically termed as ‘lichenoid GVHD,’ characterized by manifestations such as dry, scaly, and pruritic skin. In contrast, the sclerotic form may involve the dermis, subcutaneous tissue, or fascia, either with or without antecedent non-sclerotic disease. These sclerotic changes can resemble morphea, systemic sclerosis, or eosinophilic fasciitis, with tissue involvement ranging from superficial dermis to deeper fascial layers. When occurring over joints, sclerotic cGVHD often causes skin tightening, impaired mobility, and functional limitations.

## Assessing MSK GVHD

Due to the complex nature of GVHD, its evolving course, treatment-related complications, and diverse MSK presentations, concerns have been raised about inadequate supportive services, limited understanding of patient needs, and inconsistencies in physiotherapy diagnosis and management.^44,45^ cGVHD affects each patient differently, making individualized physiotherapy assessment and treatment essential. Accurately capturing functional limitations is key for monitoring MSK symptoms and guiding tailored advice on return to work, sports, ADLs, and social activities. The NIH 2014 consensus recommends the Photographic Range of Motion (P-ROM) tool, which is user-friendly, captures a wide range of function, and fits well into routine clinical practice.^46^ The P-ROM assesses four joints, namely shoulders, elbows, wrists/fingers, and ankles, using images scored from 1–7 (ankles 1–4), with lower scores indicating greater restriction and a maximum total of 25. However, it does not capture myofascial restrictions, dynamic movement in varied positions, or differentiate between isolated joint issues and global limitations from skin involvement.^47^ This has also been observed and reported in one of our previous case study publications where significant differences in wrist ROM was found when measuring the wrist with fingers closed compared to the fingers in extension.^48^ These observations and findings emphasize the need to develop a novel approach to assessing upper limb function in patients with MSK cGVHD. To help capture the true physical and functional limitations in patients and for monitoring the changes in MSK manifestations over time, the Physical Therapy Association for Graft Versus Host Disease (PTAGVHD), the Transplant Complications Working Party of the European Society for Blood and Marrow Transplantation (EBMT), the Survivorship Special Interest Group of the American Society of Blood and Marrow Transplantation (ASBMT), and the Quality of Life Committee of the Eastern Mediterranean Blood and Marrow Transplantation (EMBMT), have put forward recommendations that, where possible, clinicians should carry out a full body physical evaluation, which includes joint ROM for UL, LL, spine, alongside the strength and endurance tests.^49^

## HCT/GVHD medications and MSK manifestations

Polypharmacy significantly impacts physical assessment in HCT patients and most HCT and cGVHD patients follow complex regimens of supportive care, immunosuppressants, and antibiotics, leading to high rates of polypharmacy.^50^ Some of these medications have shown to induce musculoskeletal (MSK) symptoms, either in a dose-dependent manner or due to drug-drug interactions, a topic we have explored in much more detail in our previous publication.^51^

For example, calcineurin inhibitors (CIs) such as cyclosporine A and tacrolimus are frequently used as prophylactic treatments for cGVHD, and have shown to cause CI-induced pain syndromes and neuropathic pain in both lower extremities, allodynia, and dysesthesia.^52,53^ Similarly, drug-induced debilitating joint pain, joint necrosis, central nervous demyelination and facial nerve paresis have also been reported; however, tapering the dosage or discontinuing the medication altogether have shown to reduce or completely resolve the symptoms.^54^ A thorough knowledge of the potential MSK masquerades from drug-drug interactions or drug-induced side effects is essential for rehabilitation specialists working with this patient group, so that appropriate action can be taken if presented with such a cluster of symptoms.

## Case presentation

**Figure 1.**
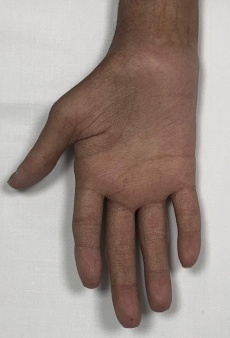
Atrophy Thenar /Hypothenar muscles

**Figure 2.**
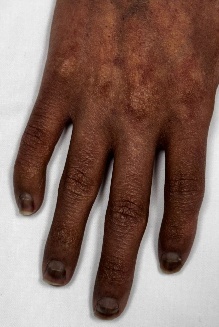
Changes in finger joints and scarring of nailbeds and matrix

**Figure 3.**
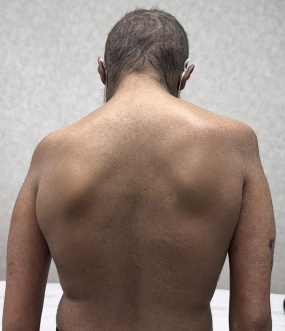
Limitation in elbow extension

**Figure 4.**
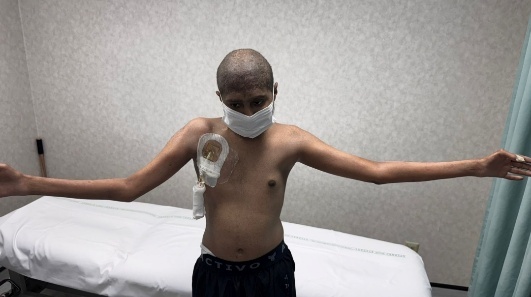
Right > Left shoulder ROM limitation

**Figure 5.**
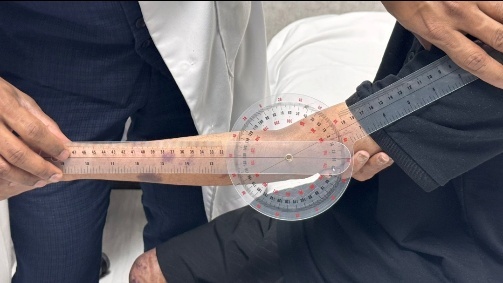
Bilateral scapular dyskinesia and mild scoliosis of spine

To provide a clearer visualization of upper limb musculoskeletal presentation in post-HCT, cGVHD patients, we have included a few images (Fig. 1-5) of an 18-year-old male patient diagnosed with Fanconi anemia and undergoing haploidentical bone marrow transplant on August 21, 2024.

This patient presented with acute and chronic GVHD overlap syndrome involving gastrointestinal, skin, and MSK affecting the upper limbs, spine, and lower limbs. He was referred to physiotherapy due to complaints of shoulder pain; however, upon conducting a comprehensive functional assessment, it was noted that he had limited range of motion in both shoulders and elbows, muscle atrophy in the thenar muscles of both hands, and early signs of joint deformities in some fingers. The case also underscores the importance of assessing limitations across all joints of the body, regardless of the target area referred for evaluation, as comprehensive full body assessment can reveal unidentified functional and strength limitations which the patient may not have recognized on their own.

## Assessment and management of MSK cGVHD manifestations

Post-transplant care often lacks integrated rehabilitation, missing early intervention opportunities. To address this, we published the first collaborative white paper from HCT groups in the USA, Europe, and the Middle East, offering comprehensive rehab guidelines for pre-, inpatient, and post-transplant care.^49^

## MSK cGVHD physiotherapy assessment

The National Institutes of Health (NIH) Consensus Criteria for Chronic Graft-versus-Host Disease (cGVHD) is the gold standard for diagnosing and grading cGVHD severity. The NIH assessment includes clinician-reported measures (CRFs), patient-reported outcomes (PROs), and objective organ scoring. Table 3 outlines the manifestations that, by themselves, are sufficient to establish the diagnosis of cGVHD, without requiring a biopsy or additional features.

**Table 3.** Diagnosis of cGVHD

Organ/System	Diagnostic Features
Skin	Poikiloderma, lichen planus-like features, sclerotic features
Mouth	Lichen planus-like changes
Eyes	New onset dry, gritty, or painful eyes with KCS (keratoconjunctivitis sicca)
GI tract	Oesophageal webs/strictures not due to other causes
Liver	Elevated bilirubin or liver enzymes with histologic confirmation
Genital tract	Lichen planus-like features (e.g., vaginal scarring)

The MSK involvement in cGVHD is distinctive but not diagnostic on its own, which means it typically requires biopsy or confirmation by other diagnostic features, such as an MRI showing fascial thickening or oedema, muscle/soft tissue biopsy confirming fasciitis or myositis (lymphocytic infiltration, fibrosis), elevated creatine kinase (CK) levels in the blood indicating skeletal muscle involvement and functional assessments such as ROM grip strength, 2-minute walk test., sit to stand etc. (Table 4).

**Table 4.** Distinctive features in MSK cGVHD

Feature	Description
Fasciitis	Inflammation and thickening of fascia; may present with tight skin, restricted movement, and pain; often involves forearms or legs.
Myositis	Inflammation of muscles; presents with weakness, muscle tenderness, or elevated muscle enzymes (e.g., CK).
Arthralgia / Arthritis	Joint pain or inflammation, with or without effusion. Joint contractures can develop over time.
Joint Stiffness / Contractures	May result from sclerotic skin changes or deeper fascial involvement. Limitation in range of motion is common.
Muscle cramps or pain	Non-specific but can occur as part of musculoskeletal GVHD.

## Pre-Transplant Physical Assessment

During the pre-HCT stage, it is recommended that, where possible, all patients undergo a full-body functional evaluation, including strength and ROM measurements of the upper and lower limbs and spine at long and short levers, also known as the kinetic chain sequence.^55^ Upper-limb strength should be recorded using JAMAR grip and pinch tests, as some studies have shown a strong correlation between hand grip strength and patients’ hospital length of stay, QoL, fatigue, and overall survival in HCT patients.^56,57^

Discussion around returning to school, sports, or work during the pre-transplant assessment might be useful for setting early goals and helping patients return to normality.

## QoL and Psychological Questionnaires

Qualitative assessment questionnaires can help explore patients’ experiences, QoL, and psychological well-being, and facilitate early identification of manifestations. Some commonly used QoL questionnaires and tools include the Functional Assessment of Cancer Therapy-Bone Marrow Transplant (FACT-BMT), the European Organization for Research and Treatment of Cancer (EORTC) QLQ-C30, and the Patient-Reported Outcomes Measurement Information System (PROMIS®).^58,59^ Additionally, Hospital Anxiety and Depression Scale (HADS), and Generalized Anxiety Disorder-7 (GAD-7) can also be used for assessing emotional distress, coping mechanisms, and mental health outcomes.^60,61^

## Management of MSK cGVHD

The management of cGVHD is primarily based on systemic therapy, with established first-line options and subsequent second- and third-line treatments available for patients with refractory or recurrent disease. Supportive care measures are integral to comprehensive management; however, they should not delay or substitute systemic therapy, which remains the cornerstone of effective cGVHD treatment.

The physiotherapy/rehabilitation management of UL MSK cGVHD-related disorders is primarily focused on exercise, stretches, and splinting, and other interventions, including manual lymph drainage (MLD) for chronic cutaneous GvHD, massage, connective tissue massage, polyneuropathy training, wraps, and light therapy with UVA A and B.^62,63^ While there are only a handful of studies on physiotherapy for scleroderma and upper limb fasciitis in cGVHD, upper limb rehabilitation specialists and hand therapists have extensive knowledge working with similar presentations in patients with systemic sclerosis (SSc), particularly with hand dysfunction, contractures, digital ulcers, and Raynaud’s phenomenon. Various non-pharmacological interventions aimed at improving hand function and QoL for SSc have been mentioned in the literature and include hand stretching exercises, ergotherapy supplemented with thermal and mud baths, whirlpool therapy, soft tissue massage, paraffin baths, McMennell joint manipulation, Maitland’s joint mobilization, and home-based rehabilitation exercises for reducing disease burden and improving QoL, with varying degrees of success.^64–66^ However, evidence-based protocols for hand therapy in upper limb cGVHD are limited due to a lack of specialized therapists working with this patient group. There is an urgent need to raise awareness among UL experts and develop standardized, targeted treatments.

## Conclusion

cGVHD is a complex condition that occurs as a side effect of allo-HCT and can have a significant physical, psychological, and socioeconomical impact upon those affected. It is essential to engage with upper limb physiotherapy and occupational therapy expert groups to raise awareness of these MSK complications, collaborate effectively to facilitate early recognition and diagnosis, and initiate tailored, evidence-based management to optimise patient outcomes.

### Authors’ Contribution per CRediT

Jaleel A. Mohammed – Conceptualization, Data curation, Methodology, Project administration, Resources, Writing – original draft

Caroline Couthard – Formal Analysis, Methodology, Validation, Writing – review & editing

Ahmed AlGhamdi – Writing – review & editing

Collins Ogbeivor - Writing – review & editing

Shahrukh K. Hashmi - Formal Analysis, Supervision, Writing – review & editing

### Conflict of Interest – COPE

No competing interests were disclosed.

### Ethical Conduct Approval – Helsinki – IACUC

The King Faisal Specialist Hospital & Research Centre Ethics Committee approved this study (Study Number: 2251327, 18/06/2025). Informed consent was obtained from the participant for the use of pics for publication.

### Informed Consent Statement

All authors and institutions have confirmed this manuscript for publication.

## Supplementary Material

Supplementary Supplement / Search methodology

## Data Availability

All are available upon reasonable request.
