# Supplementary material for: Chronic graft versus host disease in upper limb: Calling upper limb rehabilitation specialists for improving outcomes in patients
Source: Clin Hematol Int. 2026 Mar 11;8(1):37–48. doi: 10.46989/001c.158500 (PMC12982154; doi:10.46989/001c.158500)
Supplement: Supplementary — Supplement / Search methodology [file chi_2026_8_1_158500_333385.pdf]

Supplement / Search methodology

EMBASE/MEDLINE	Articles
hematopoietic AND ('stem'/exp OR stem) AND ('cell'/exp OR cell) AND ('transplantation'/exp OR transplantation) AND ('physiotherapy'/exp OR physiotherapy) AND [1990-2025]/py AND ('article'/it OR 'review'/it OR 'clinical trial'/it) AND [english]/lim AND ([embase]/lim OR [medline]/lim)	241
acute AND graft AND versus AND host AND disease AND physiotherapy AND [1990-2025]/py AND [english]/lim AND [humans]/lim AND ([embase]/lim OR [medline]/lim)	63
('chronic graft versus host disease'/exp OR 'chronic graft versus host disease') AND ('physiotherapy'/exp OR 'physiotherapy') AND musculoskeletal AND [english]/lim AND [humans]/lim AND ([embase]/lim OR [medline]/lim) AND [1990-2025]/py	13
'chronic graft versus host disease' AND fasciits AND [english]/lim AND [humans]/lim AND ([embase]/lim OR [medline]/lim) AND [1990-2025]/py	0
'chronic graft versus host disease' AND scleroderma AND [english]/lim AND [humans]/lim AND ([embase]/lim OR [medline]/lim) AND [1990-2025]/py	449
'chronic graft versus host disease' AND rehabilitation AND [english]/lim AND [humans]/lim AND ([embase]/lim OR [medline]/lim) AND [1990-2025]/py	154
**Total**	920

Web Science	Articles
Hematopoietic stem cell transplantation (Topic) AND physiotherapy	62
Hematopoietic stem cell transplantation (Topic) AND musculoskeletal	298
Hematopoietic stem cell transplantation (Topic) AND fasciitis	47
Hematopoietic stem cell transplantation (Topic) AND scleroderma	609
Acute graft versus host disease (Topic) AND fasciitis	29
Chronic graft versus host disease (Topic) AND fasciitis	77
Chronic graft versus host disease (Topic) AND Musculoskeletal	91
Acute graft versus host disease (Topic) AND Musculoskeletal	44
Acute graft versus host disease (Topic) AND scleroderma	15
Chronic graft versus host disease (Topic) AND scleroderma	509
chronic graft versus host disease (Topic) AND physiotherapy (Topic) AND musculoskeletal	77
Acute graft versus host disease (Topic) AND physiotherapy (Topic) AND musculoskeletal	4
Hematopoietic stem cell transplantation (Topic) AND physiotherapy (Topic) AND musculoskeletal	4
Bone Marrow Transplant (Topic) AND physiotherapy (Topic) AND musculoskeletal	1
Stem Cell Transplant (Topic) AND physiotherapy (Topic) AND musculoskeletal	0

Chronic graft versus host disease (Topic) AND physiotherapy (Topic) AND scleroderma	1
Acute graft versus host disease (Topic) AND physiotherapy (Topic) AND scleroderma	1
Total	1869

PubMed	Articles
(((graft versus host disease) OR (chronic graft versus host disease)) OR (acute graft versus host disease)) AND (physiotherapy)	11
(((graft versus host disease) OR (chronic graft versus host disease)) OR (acute graft versus host disease)) AND (musculoskeletal)	21
(((graft versus host disease) OR (chronic graft versus host disease)) OR (acute graft versus host disease)) AND (scleroderma)	13
(((graft versus host disease) OR (chronic graft versus host disease)) OR (acute graft versus host disease)) AND (musculoskeletal complications)	8
(((graft versus host disease) OR (chronic graft versus host disease)) OR (acute graft versus host disease)) AND (skin)	3
((((Hematopoietic Cell Transplant) OR (Hematopoietic stem Cell Transplant)) OR (Stem Cell Transplant)) OR (Bone Marrow Transplant)) AND (musculoskeletal)	111
((((Hematopoietic Cell Transplant) OR (Hematopoietic stem Cell Transplant)) OR (Stem Cell Transplant)) OR (Bone Marrow Transplant)) AND (physiotherapy)	163
((((Hematopoietic Cell Transplant) OR (Hematopoietic stem Cell Transplant)) OR (Stem Cell Transplant)) OR (Bone Marrow Transplant)) AND (rehabilitation)	457
((((Hematopoietic Cell Transplant) OR (Hematopoietic stem Cell Transplant)) OR (Stem Cell Transplant)) OR (Bone Marrow Transplant)) AND (physiotherapy assesment)	0
((((Hematopoietic Cell Transplant) OR (Hematopoietic stem Cell Transplant)) OR (Stem Cell Transplant)) OR (Bone Marrow Transplant)) AND (physical assesment)	0
(Hematopoietic Cell Transplant) AND (upper limb specialist)	0
(graft versus host disease) AND (upper limb specialist)	0
Total	787

Scopus	Articles
haematopoietic AND stem AND cell AND transplantatio OR stem AND cell AND transplant OR bone AND marrow AND transplant AND physiotherapy	422
haematopoietic AND stem AND cell AND transplantation AND physiotherapy AND scleroderma	25
haematopoietic AND stem AND cell AND transplantation AND physiotherapy AND skin	94
haematopoietic AND stem AND cell AND transplantation OR stem AND cell AND transplant OR bone AND marrow AND transplant AND physiotherapy AND skin	111
haematopoietic AND stem AND cell AND transplantation OR stem AND cell AND transplant bone AND marrow AND transplant AND physiotherapy AND musculoskeletal	137
haematopoietic AND stem AND cell AND transplantation OR stem	28

AND cell AND transplant<br>OR bone AND marrow AND transplant AND physiotherapy<br>AND scleroderma	
chronic AND graft AND versus AND host AND disease<br>AND upper AND limb AND specialist	19
Total	836
